# Early nourishment, better survival: association between breastfeeding initiation and infant mortality in Indian tribes

**DOI:** 10.1186/s12889-025-23084-6

**Published:** 2025-05-23

**Authors:** Mohammad Hammad, Mohammad Hifz Ur Rahman

**Affiliations:** 1 Manipal Tata Medical College, Manipal Academy of Higher Education, Manipal, India; 2 Department of Public Health, College of Medicine and Health Sciences, National University of Science and Teachnology, Sohar, Oman

**Keywords:** Breastfeeding, Mortality, Child, India, Indigenous

## Abstract

**Background:**

Timely breastfeeding initiation within one hour of birth is recommended to reduce neonatal and early infant mortality. However, rates of early breastfeeding remain suboptimal in India, especially among marginalized tribal communities, which continue to experience disproportionately high infant mortality. The study investigated the association between the late breastfeeding initiation and infant mortality among the tribal population in India.

**Method:**

The study utilized data from the fifth round if the National Family Health Survey, which provided a sample of 232,920 most recent live births in the past five years with data on breastfeeding initiation time and infant mortality. Associations between late initiation (> 1 hour) and mortality were analysed using Cox proportional hazards regression and Kaplan-Meier survival curves.

**Results:**

The results showed that infants breastfed after the first hour of life had a 30% higher risk of infant mortality compared to those breastfed within an hour of birth (aHR: 1.30, 95% CI: 1.06–1.60). The Kaplan-Meier curves further highlighted the lower chances of survival when breastfeeding was delayed.

**Conclusion:**

These findings underscore the need for promoting early breastfeeding initiation through culturally appropriate interventions in tribal areas as a strategy to reduce persistent child survival disparities in India.

## Introduction

Breastfeeding is the natural and optimal way of feeding infants and young children. It provides them with the essential nutrients and antibodies that protects against various diseases and infections. Breastfeeding also offers benefits for the mother, such as reducing the risk of breast and ovarian cancer and promoting maternal-infant bonding [1]. Moreover, breastfeeding is also one of the most effective ways to reduce infant mortality, especially in the first month of life. The World Health Organization (WHO), initiating breastfeeding within an hour of birth to provide optimal nutrition, immunity, and bonding for the newborn, offering protection against infections, malnutrition, and other causes of mortality [2, 3]. Recent estimates from a series of papers in the Lancet indicate that breastfeeding could potentially prevent 800,000 deaths (13%) each year in developing countries. The series of papers also emphasizes that children who are exclusively breastfed have a 14-fold lower risk of dying within the first six months of life [4]. These statistics underscore the critical role of breastfeeding in global development initiatives aimed at fostering a healthier, wealthier, and more sustainable planet.

Breastfeeding aligns with various Sustainable Development Goals (SDGs) within the 2030 agenda for sustainable development [4]. Goals 2 and 3 which focus on hunger, health, and well-being. are directly supported by breastfeeding as a crucial nutritional source capable of saving children’s lives and fostering enhanced health outcomes for both children and mothers [4]. Goals 1, 8, and 10 which target the eradication of poverty, stimulation of economic growth, and mitigation of inequalities are also impacted by breastfeeding. Studies have shown that breastfeeding contributes approximately US$302 billion each year as supplementary income to the global economy, representing nearly 0.5% of the world’s gross national product [5]. Furthermore, Goal 4 which focuses on education is supported by the correlation between breastfeeding and elevated IQs, as well as improved educational achievements [4, 6–8].

However, in India, breastfeeding rates remain suboptimal and vary significantly across different states and districts. According to the National Family Health Survey (NFHS– 5) for 2019-20, only 64% of children (0–6 months) are exclusively breastfed, and only 42% of children are breastfed within one hour of birth [9, 10]. While breastfeeding is a widespread practice among Indian mothers, the initiation of breastfeeding often occurs later than recommended, and colostrum is frequently discarded. In rural areas, breastfeeding practices are shaped by local beliefs, which, are influenced by a combination of social, cultural, and economic factors [11–16].

Breastfeeding practices among tribal mothers in India exhibit unique characteristics influenced by cultural beliefs, economic factors and healthcare access. Interestingly, breastfeeding initiation is often earlier in tribal mothers as compared to their non-tribal counterparts [17].

A cross-sectional study found that the exclusive breastfeeding (EBF) rate among tribal mothers is relatively high. However, the overall EBF rate for the first six months showed significant regional variations [18]. The National Family Health Surveys (NFHS) 4 and 5 indicated that infants of scheduled tribes have higher rates of being exclusively breastfed at six months compared to other groups [18].

Despite these positive trends, certain practices may negatively impact infant health. Pre-lacteal feeding, where infants are given substances other than breast milk before breastfeeding is initiated, has been observed among tribal mothers [19]. Additionally, despite the recognized health benefits of colostrum, in some cases, mothers do not provide this nutrient-rich first milk to their newborns [19]. These practices may contribute to adverse health outcomes for infants in tribal communities.

Tribal groups have historically endured social marginalization and inadequate health infrastructure in India, reflected in disproportionately high child mortality burdens [13]. The infant mortality rate among scheduled tribes remains high at 41.6 per 1,000 live births compared to the national average of 35.2, as per NFHS-5 data. Despite the unique breastfeeding patterns observed in tribal communities, they continue to face disproportionately high infant mortality rates. A study by Niswade et al. (2011) found that neonatal mortality rates among tribal populations were 39% higher than non-tribal populations, even after adjusting for socioeconomic factors [20]. This persistent disparity highlights the need for targeted interventions and a deeper understanding of the factors influencing infant survival in these communities.

While several small-scale studies have examined breastfeeding practices in specific tribal groups, there remains a notable gap in comprehensive, national-level analyses of how these practices relate to infant mortality outcomes across India’s diverse tribal populations. This lack of large-scale quantitative evidence specifically delineating the linkage between suboptimal breastfeeding and elevated child mortality risks faced by tribal populations in India presents a significant research opportunity.

In 2016, the Indian government launched the “Mothers Absolute Affection” (MAA) program to promote breastfeeding. Despite significant efforts by healthcare practitioners in India to encourage breastfeeding, the country’s breastfeeding rate still falls short of the World Health Organization’s targeted goal of 50%. As far as our knowledge extends, there is a lack of comprehensive national-level studies in India addressing suboptimal breastfeeding practices within tribal communities, including delayed initiation among children.

This study aims to address gap by investigating the association between delayed breastfeeding initiation and infant mortality among tribal communities on a national scale. By doing so, we intend to provide crucial evidence to inform targeted public health interventions and policies, ultimately contributing to the reduction of health disparities and improvement of infant survival rates in these vulnerable populations.

## Methods

### Data

Our analysis is based on the data sourced from the National Family Health Survey-5 (NFHS-5) conducted in India from 2019 to 2021. The National Family Health Surveys (NFHS) constitute a series of cross-sectional, nationally representative surveys designed to capture information on various demographic, socioeconomic, maternal, and child health indicators, as well as reproductive health and family planning aspects. Employing a two-stage stratified sampling approach, the fifth round of NFHS involved interviewing approximately 724,115 women aged 15 to 49 from 636,699 households, achieving a commendable 92% response rate. Additional details regarding the sampling strategy and tools employed can be utilized elsewhere [10]. Kid’s Recode file (IAKR7EFL) was utilized for the analysis which has a sample size of 232,920 consisting of data of births to women aged 15–49 years in the preceding 5 years. After limiting the data to only tribal women and cleaning the data the study sample was 10,633 which consisted of births to the tribal women aged 15–49 years in the past 5 years preceding the survey.

### Preliminary definitions

#### Outcome variable

The primary outcome variable of the study was infant mortality i.e. the death of the child before their first birthday. It was coded as ‘0’ meaning ‘survived’ and ‘1’ meaning ‘didn’t survive’.

#### Independent variables

The primary variable under examination was the initiation time of breastfeeding, categorized into two groups: within one hour (early initiation) and after one hour (late initiation), as reported by the mothers based on their recall. Sociodemographic attributes encompassed the mother’s age, educational level, wealth index, and place of residence. Reproductive characteristics included birth order, while child factors comprised the size and sex of the child at birth.

### Statistical analysis

Descriptive statistics were calculated for sample characteristics. The relationship between late breastfeeding initiation and infant mortality was modelled using Cox proportional hazards regression. Hazard ratios (HR) with 95% confidence intervals (CI) were computed adjusting for relevant confounding variables.

$$\:h\left(t\right)=\:{h}_{0}\left(t\right)\text{exp}\left({\beta\:}_{1}{X}_{1}+\:{\beta\:}_{2}{X}_{2}+\:\dots\:+\:{\beta\:}_{n}{X}_{n}\right)$$

Kaplan-Meier survival curves demonstrated the probability of infant survival over time by breastfeeding initiation categories. All analyses were conducted using Stata version 16.0, accounting for the complex survey design of the NFHS-5 through appropriate survey weights and clustering adjustments.

## Results

Table 1 gives the sample characteristics of tribal children along with the background characteristics. 78.8% of children were breastfed within 1 hour of the birth. A significant majority of the children belonged to the rural areas (89.9%). Maternal characteristics showed most of the children were born to women in the age group of 21–30 years (65.6%). Only 7% of mothers had attained till the higher level and one-fourth of the mothers were illiterate. The data also shows an equivalent amount of distribution of boys and girls across the nation.

**Table 1:** Sample characteristics of tribal children by demographic, socio-economic, maternal and child factors, NFHS-5 (2019-21)

Variables	Frequency	Percent
**Time to breastfed after birth**		
Within 1 hour	8,374	78.75
After 1 hour	2,259	21.25
**Place of Residence**		
Urban	1,064	10.01
Rural	9,569	89.99
**Wealth index**		
Poorest	5,213	49.03
Poorer	2,741	25.78
Middle	1,527	14.36
Richer	812	7.64
Richest	340	3.2
**Mother’s Education**		
No education	2,799	26.32
Primary	1,661	15.62
Secondary	5,360	50.41
Higher	813	7.65
**Age of women at birth**		
20 yrs & below	1,525	14.34
21–30 yrs	6,974	65.59
30 yrs & above	2,134	20.07
**Number of antenatal visits during pregnancy**		
No visits	1,955	18.39
1 to 4 visits	5,068	47.66
More than 4 visits	3,610	33.95
**Body mass index of women**		
Thin	2,128	20.35
Normal	7,240	69.23
Overweight	921	8.81
Obese	169	1.62
**Birth Interval**		
< 2 yrs	1,807	26.78
2–3 yrs	1,924	28.52
> 3 yrs	3,016	44.7
**Birth order of the child**		
1–2	6,583	61.91
3+	4,050	38.09
**Size of the child at birth**		
Large	1,850	18.01
Medium	7,299	71.04
Small	1,125	10.95
**Sex of the child**		
Male	5,474	51.48
Female	5,159	48.52
**N**	**10**,**633**	

**Fig. 1:**
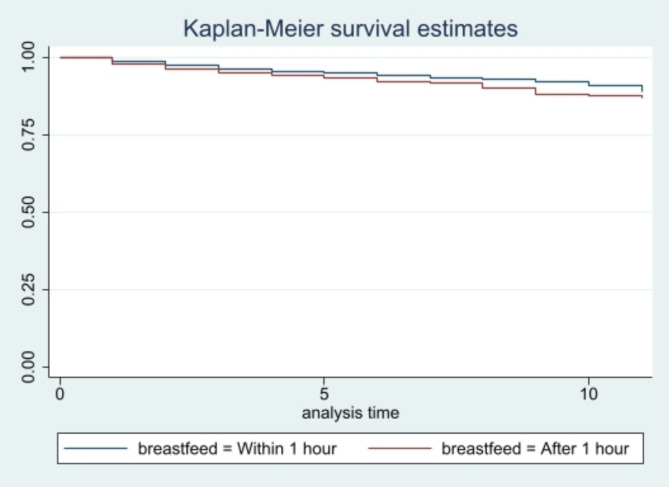
Kaplan-Meier survival curve for infant survival according to the time of initiation of breastfeeding

Figure 1 plot shows the Kaplan-Meier survival estimates for the survival of child till the first year of birth with breastfeeding within one hour of birth as factor variable. The figure illustrates that 12.1% of the children died before celebrating their first birthday who were breastfed after one hour from the time of their birth.

Table 2 shows the infant mortality per 1000 livebirths by the key explanatory variables considered in the study. The data shows that infant mortality rates are significantly lower for infants who were breastfed within an hour of their birth (30.4 per 1000 live births) as compared to the infants who were breastfed after an hour of their births (72.6 per 1000 live births). The rates also showed a significant difference in urban areas (29.1 per 1000 live births) compared to rural areas (41.0 per 1000 live births), There is a clear pattern where infant mortality rates decrease as wealth increases. The poorest households have the highest mortality rate (44.6 per 1000 live births), while the richest households have the lowest mortality rate (21.0 per 1000 live births) similar to wealth, there is an inverse relationship between education level and infant mortality. Higher education levels correspond to lower mortality rates (25.1 per 1000 live births) in comparison to those who are un-educated (45.7 per 1000 live births).

**Table 2:** Infant mortality rate (per 1000 live births) by demographic, socio-economic, maternal and child factors, NFHS-5 (2019-21)

Infant mortality	Level	Infant Mortality Rate (per 1000 live births)
**Time to breastfed after birth**	Within 1 hour	30.4
	After 1 hour	72.6
**Place of residence**	Urban	29.1
	Rural	41.0
**Wealth index**	Poorest	44.6
	Poorer	41.0
	Middle	33.9
	Richer	24.6
	Richest	21.0
**Mother’s Education**	No education	45.7
	Primary	41.1
	Secondary	37.1
	Higher	25.1
**Age of women at birth**	20 yrs & below	48.7
	21–30 yrs	36.9
	30 yrs & above	42.1
**Number of antenatal visits during pregnancy**	No visits	70.5
	1 to 4 visits	26.7
	More than 4 visits	23.9
**Birth Interval**	< 2 yrs	46.4
	2–3 yrs	33.0
	> 3 yrs	33.8
**Birth order of the child**	1–2	30.7
	3+	53.4
**Sex of child**	Male	42.1
	Female	37.3

Table 3 explains the risk of infant mortality with the primary variable under examination initiation of time of breastfeeding after birth of the child. The delay in initiation of breast feeding beyond an hour after birth of child by the mothers increases the risk of infant mortality by 30% (HR 1.30 99% CI: 1.06,1.60) among the newborns.

**Table 3:** Cox-hazard model depicting risk of infant mortality according to the time of initiation of breastfeeding

Variable	Hazard Ratio	*p*-value	95% Confidence Interval	
**Time to breastfed after birth**				
Within 1 hour (Ref.)				
After 1 hour	1.303	0.011	1.062	1.600
**Place of residence**				
Urban (ref.)				
Rural	1.064	0.741	0.735	1.540
**Wealth index**				
Poorest (ref.)				
Poor	1.065	0.557	0.861	1.317
Middle	1.095	0.544	0.816	1.470
Rich	0.988	0.959	0.626	1.557
Richest	0.988	0.975	0.478	2.039
**Mother’s education**				
No education (Ref.)				
Primary	0.818	0.118	0.637	1.051
Secondary	0.694	0.001	0.560	0.861
Higher	0.561	0.032	0.331	0.950
**Age of women at birth**				
Below 20 years (Ref.)				
21–30 yrs	0.515	0.000	0.399	0.666
30 yrs & above	0.328	0.000	0.234	0.458
**Birth order of the child**				
1–2 (ref.)				
3+	2.924	0.000	2.374	3.601
**Weight of child at birth**				
Underweight (ref.)				
Normal	0.560	0.000	0.446	0.703
Above normal	0.377	0.000	0.278	0.509
Not weighted	0.908	0.488	0.691	1.192
**Size of child at birth**				
Large (ref.)				
Medium	0.980	0.873	0.775	1.241
Small	1.250	0.158	0.916	1.705
**Sex of the child**				
Male (ref.)				
Female	0.808	0.016	0.679	0.961

Among the socio demographic factors, the risk of mortality lessens significantly with the education of the mother as compared to mother having no education at all with the risk being 44% lesser in women who have received education till the higher level (HR 0.56 95% CI: 0.33, 0.95) and being 31% lesser in women who have received education till the secondary level (HR 0.69 99% CI: 0.56,0.86). The age of the mother has also a significant impact on the survival of the child with older mothers being less prone to suffer infant mortality (HR 0.32 99% CI: 0.23,0.45) as compared to adolescent mothers but the birth order of the as it increases poses a great risk of child not surviving till their first birthday the children with 3 + birth order have a very high likelihood of mortality in their infancy (HR 2.92 99% CI: 2.37,3.60). Infants with normal or above-normal birth weight have a lower risk of mortality before their first birthday. Smaller birth size is associated with a 25% higher risk of infant mortality (HR 1.25, 95% CI: 0.91, 1.71), though this was not statistically significant in our model. The female baby has a higher likelihood of survival (HR 0.80 95% CI: 0.67, 0.96) as compared to a male baby in their infancy.

## Discussion

The current study emphasizes the significant effect of early initiation of breastfeeding on infant mortality among tribal communities in India. Our findings indicate that delayed breastfeeding initiation beyond one hour after birth is associated with a 30% increase in the risk of infant mortality. This result is particularly significant given the context of tribal health in India, where populations often face multiple health disparities and challenges in accessing healthcare services.

Tribal populations in India are often isolated, concentrated in certain regions, and live in hilly and forested areas that make communication and access to services difficult even in normal circumstances. These geographical and social barriers contribute to the higher infant mortality rates observed in tribal populations compare to non-tribal populations. Our study provides a rare systemic examination of factors associated with infant mortality in these vulnerable communities, addressing a critical gap in the literature The importance of early initiation of breastfeeding in reducing infant mortality is well established, but its specific impact in tribal communities has been understudied. Our findings align with previous research showing that early initiation of breastfeeding can prevent around 20% of neonatal deaths. However tribal context presents unique challenges, many studies have shown that tribal women often do not initiate breastfeeding within the first hour of birth and some squeeze out the first milk before initiating breastfeeding [19–22]. These practices, likely rooted in cultural beliefs and traditions, may contribute to the higher infant mortality rates observed in these communities.

The timing of breastfeeding initiation is a critical factor in infant survival. Early initiation of exclusive breastfeeding marks the beginning of a continuum of care for both mother and newborn, with long-lasting effects on health and development [11, 17, 18]. Our study quantifies this impact, showing that delayed initiation increases mortality risk by 30%, a finding that has significant implications for public health interventions in tribal areas.

Socio-economic factors play a crucial role in infant mortality, as evidenced by our results showing an inverse relationship between wealth and mortality rates. This is consistent with previous literature indicating that Infant mortality rates are inversely related to socio-economic status [23–25].

The disparities we observed based on place of residence (urban vs. rural) and maternal education level further underscore the complex interplay of social determinants of health in tribal communities.

The Maternal factors, including age and education, emerged as significant predictors of infant survival in our study. This aligns with existing literature on the influence of maternal age on birth and infant outcomes [26–28]. The higher risk of mortality associated with higher birth order (3 + births) in our study highlights the need for targeted interventions for multiparous women in tribal communities.

The gender difference in infant survival observed in our study, with female infants having a higher likelihood of survival, is an interesting finding that warrants further investigation. This contrasts with some previous studies in India that have found higher mortality rates among female infants [29, 30], suggesting that gender dynamics in tribal communities may differ from those in the general population.

Our study has some limitations, including the self-reported nature of the NFHS-5 data and the potential for recall bias. Moreover, our study did not examine the relationship between place of birth (hospital vs. home) and early breastfeeding initiation. Given the unique healthcare access challenges faced by many tribal communities, this factor could play a significant role in breastfeeding practices and outcomes. We recommend that future studies specifically investigate this relationship to provide a more comprehensive understanding of the factors influencing early breastfeeding initiation and infant mortality in tribal populations. However, the large sample size and the focus on tribal populations provide valuable insights into a traditionally understudied group.

Our findings highlight the critical importance of promoting early breastfeeding initiation in tribal communities as a strategy to reduce infant mortality. The 30% increase in mortality risk associated with delayed initiation underscores the potential impact of targeted interventions. Future public health efforts should focus on culturally appropriate strategies to encourage optimal breastfeeding practices, particularly targeting less educated mothers and those with higher-order births. Additionally, addressing the broader socioeconomic determinants of health in tribal communities will be crucial for improving overall child health outcomes.

## Conclusion

This large-scale study provides strong evidence that delayed breastfeeding initiation beyond one hour of birth is associated with a 30% higher risk of infant mortality among infants born in socioeconomically deprived tribal communities in India. Additionally, we found that maternal education, birth order, and infant weight at birth were significant factors influencing infant survival. These findings highlight the critical importance of promoting early breastfeeding initiation in tribal areas. Future interventions should focus on culturally appropriate strategies to encourage optimal breastfeeding practices, particularly targeting less educated mothers and those with higher-order births. While our study demonstrates the potential impact of early breastfeeding on reducing infant mortality, further research is needed to understand the complex interplay of factors affecting infant health in these vulnerable populations.

## Data Availability

The data that supports the findings of this study are available on request. The dataset used in the study is available in the public domain and can be accessed on a request from DHS at https://dhsprogram.com/Data/. Dataset and materials used in this study are available on request from the corresponding author mohammad.rahman@manipal.edu.
